# Supplementary material for: Synthesis of Novel 2-(Cyclopentylamino)thiazol-4(5H)-one Derivatives with Potential Anticancer, Antioxidant, and 11β-HSD Inhibitory Activities
Source: Int J Mol Sci. 2023 Apr 14;24(8):7252. doi: 10.3390/ijms24087252 (PMC10139140; doi:10.3390/ijms24087252)
Supplement: Supplementary file 1 [file ijms-24-07252-s001.zip › ijms-2319803-supplementary.pdf]

Supplementary information

Novel 2-(cyclopentylamino)thiazol-4(5*H*)-one derivatives – synthesis and screening for their anticancer, antioxidantand and 11β-HSD inhibitory activities

Szymon Baumgart[1]*, Daria Kupczyk[2], Aneta Archała[3], Oliwia Koszła[3], Przemysław Sołek[3], Wojciech Płaziński[3,4], Anita Płazińska[3] and Renata Studzińska[1,*]

[1] Nicolaus Copernicus University in Toruń, Collegium Medicum in Bydgoszcz, Faculty of Pharmacy, Department of Organic Chemistry, 2 Jurasza Str., 85–089 Bydgoszcz, Poland; sz.baumgart@cm.umk.pl, rstud@cm.umk.pl

[2] Nicolaus Copernicus University in Toruń, Collegium Medicum in Bydgoszcz, Faculty of Medicine, Department of Medical Biology and Biochemistry, 24 Karłowicza Str., 85–092 Bydgoszcz, Poland; dariak@cm.umk.pl

[3] Department of Biopharmacy, Medical University of Lublin, 4a Chodźki Str., 20-093 Lublin, Poland; anita.plazinska@umlub.pl, aneta.banach94@o2.pl, koszlaoliwia@gmail.com, pp.solek@gmail.com

[4] J. Haber Institute of Catalysis and Surface Chemistry, Polish Academy of Sciences, 8 Niezapominajek Str., 30-239 Cracow, Poland; wojtek_plazinski@tlen.pl

* Correspondence: rstud@cm.umk.pl, sz.baumgart@cm.umk.pl

<u>S1. ^{1}H NMR spectra of compounds **3a** – **3i**</u>

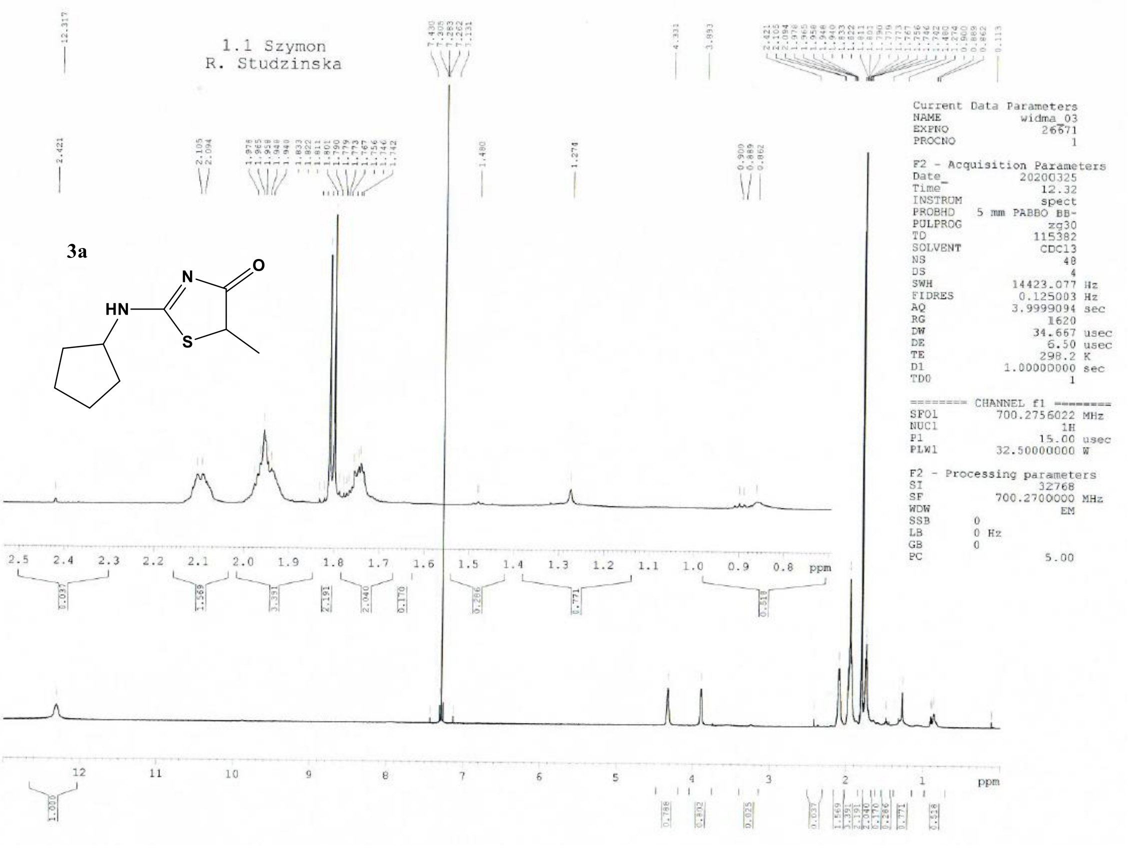

Figure S1. ^{1}H NMR spectra of compound **3a**

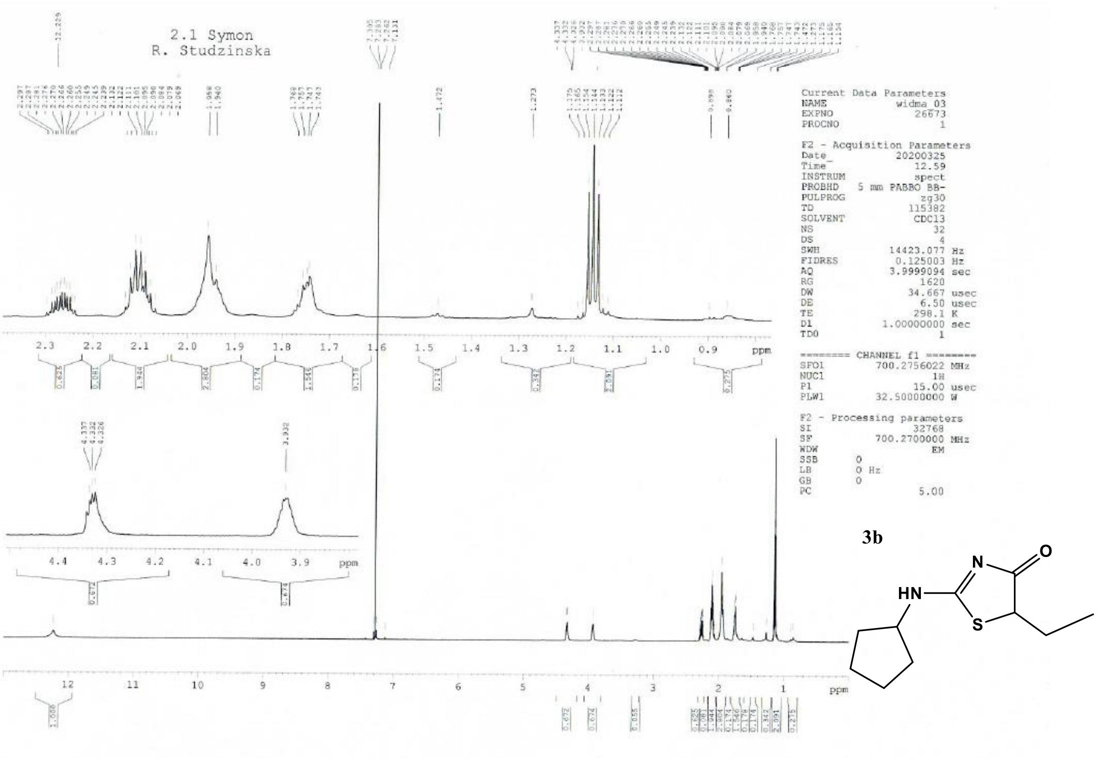

Figure S2. ^{1}H NMR spectra of compound **3b**

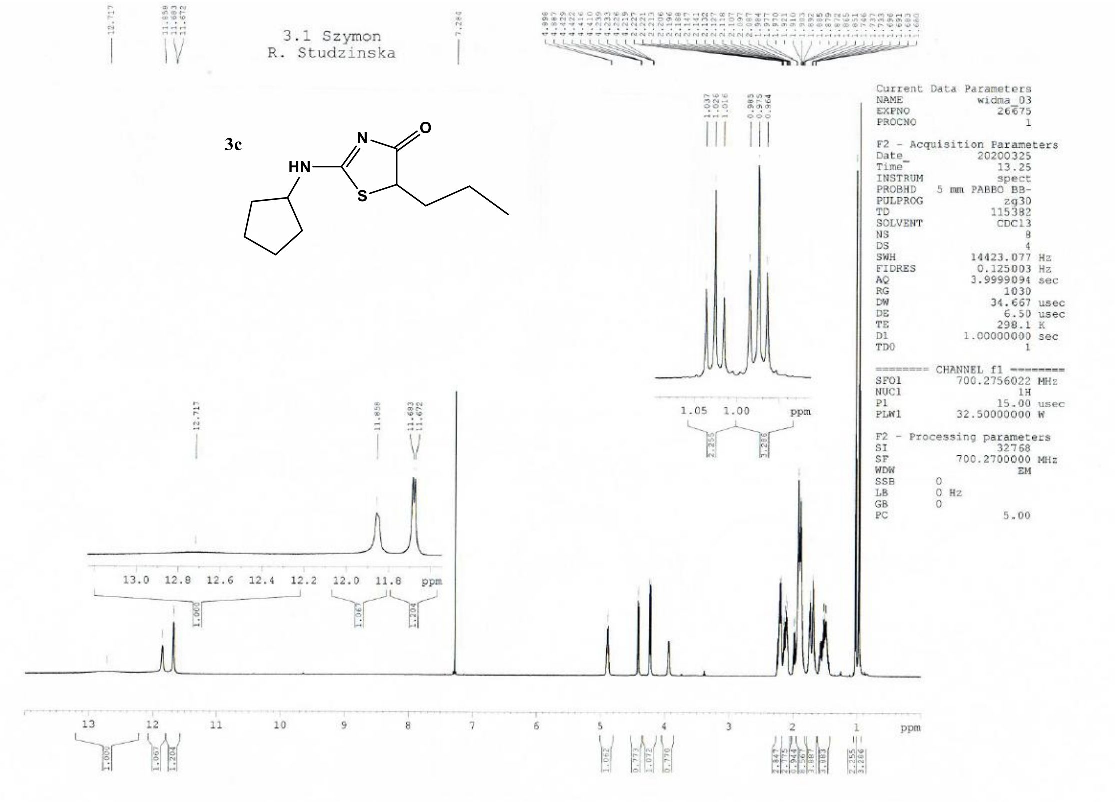

Figure S3. ^{1}H NMR spectra of compound **3c**

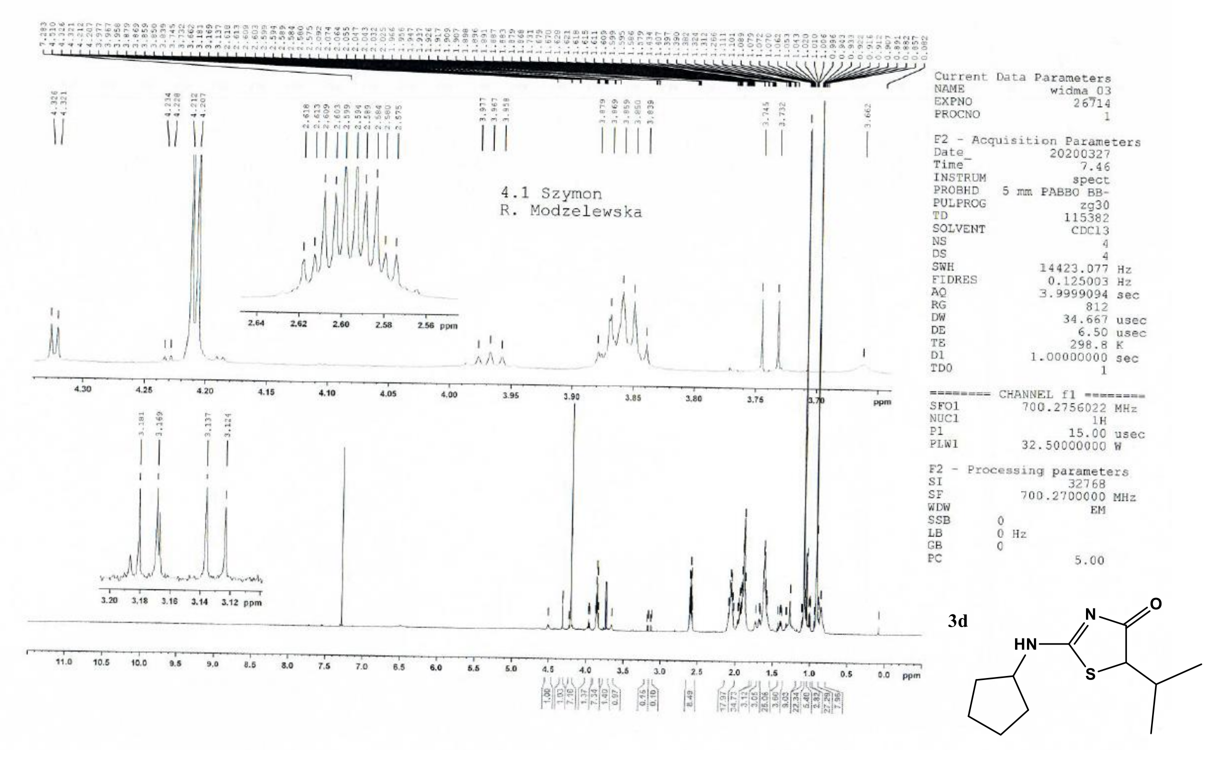

Figure S4. ^{1}H NMR spectra of compound **3d**

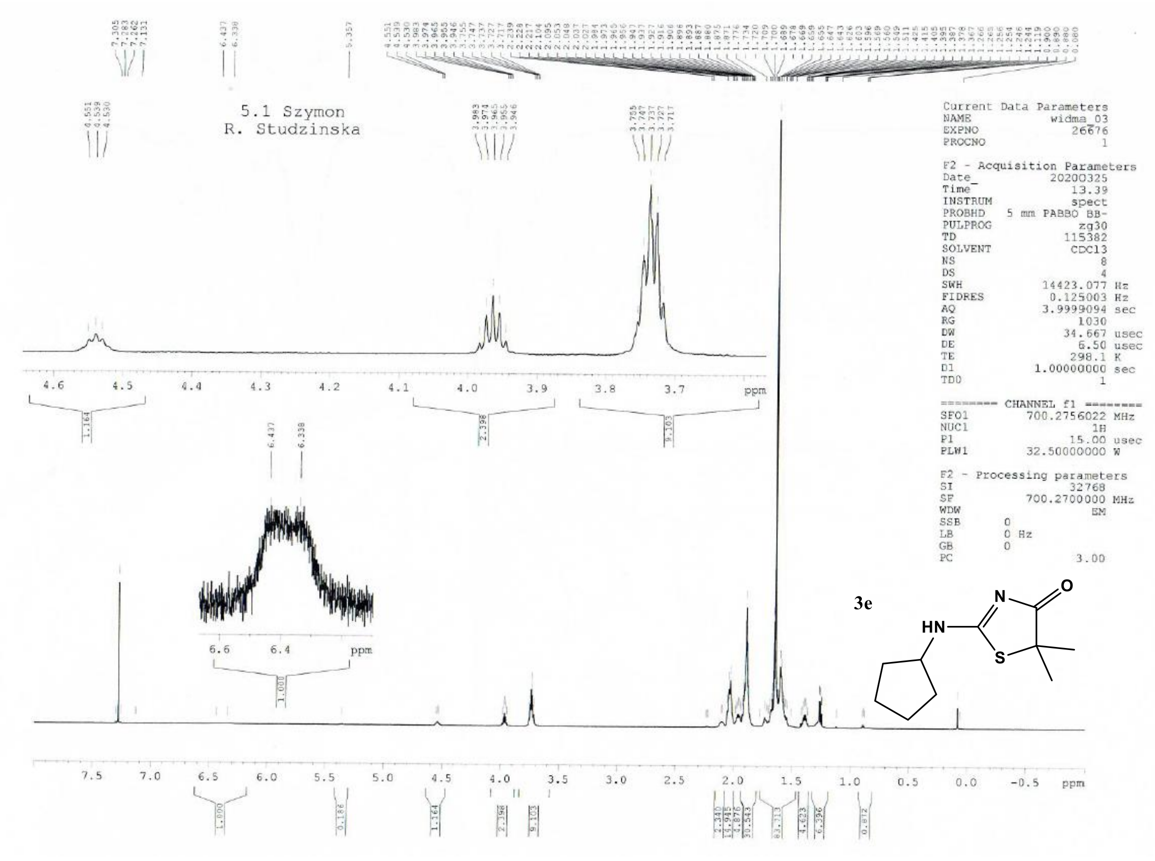

Figure S5. ^{1}H NMR spectra of compound **3e**

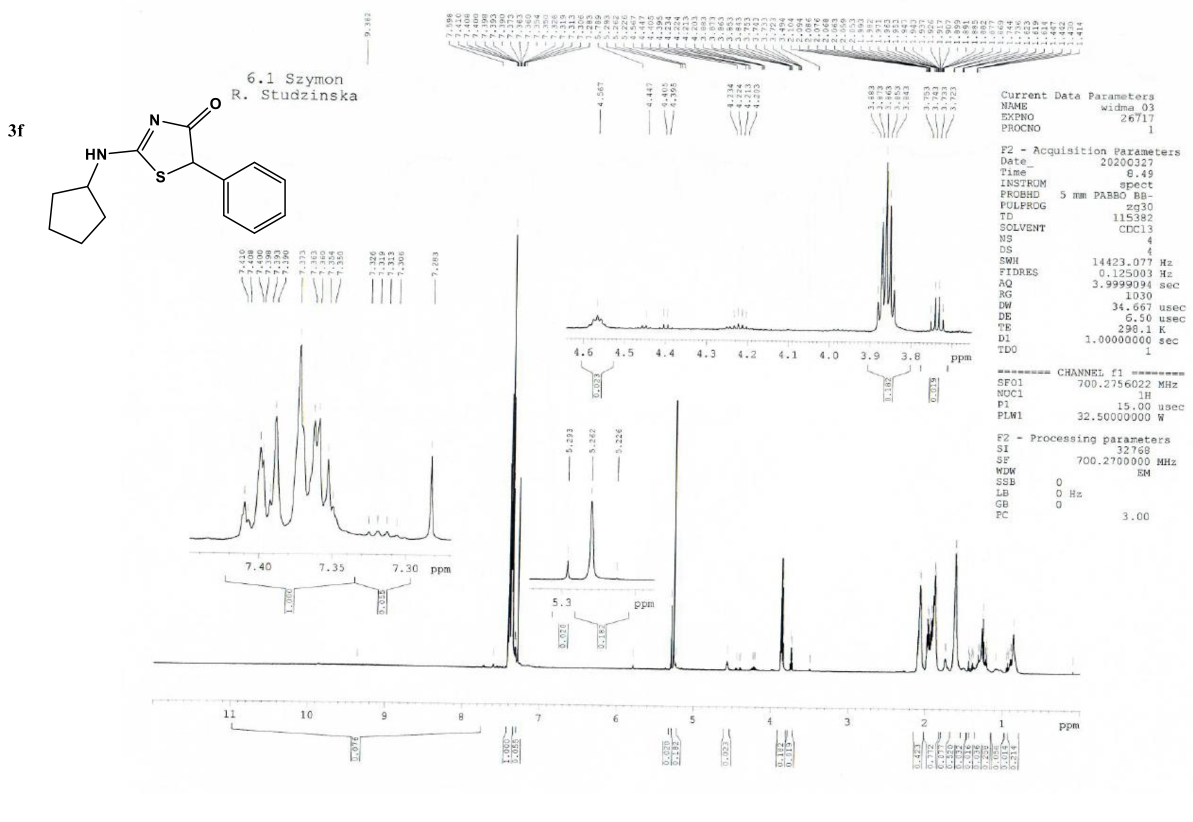

Figure S6. ^{1}H NMR spectra of compound **3f**

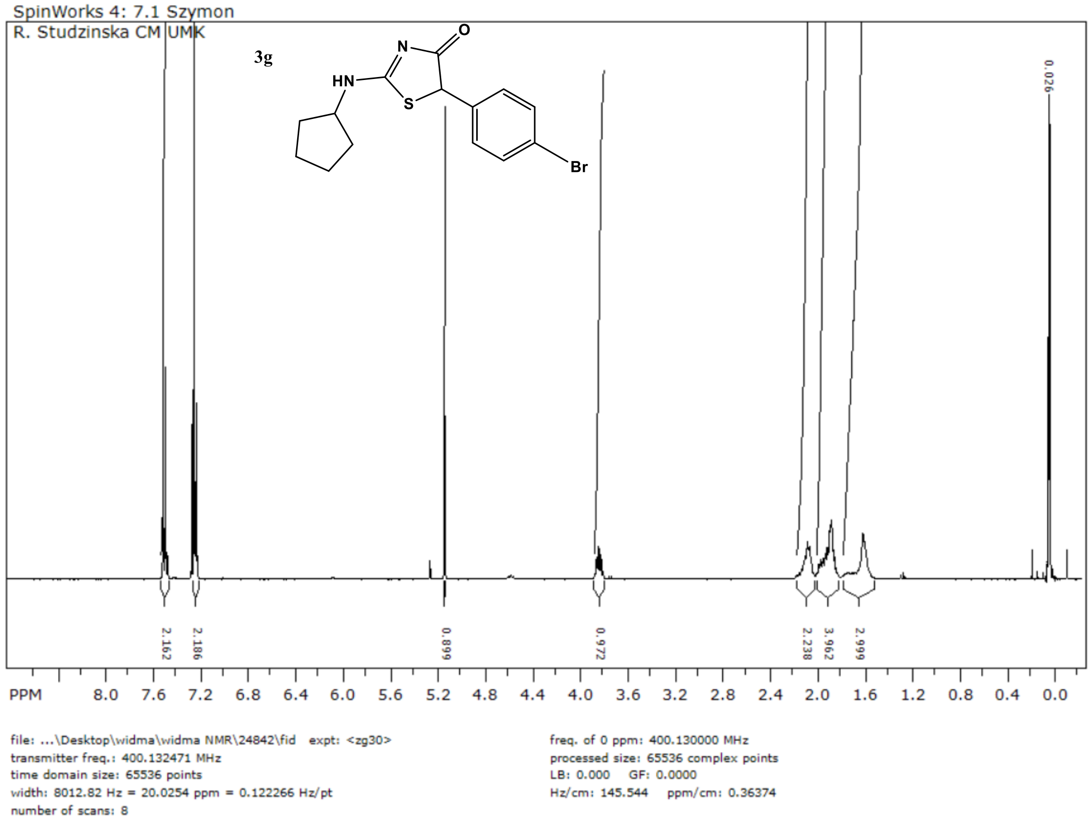

Figure S7. ^{1}H NMR spectra of compound **3g**

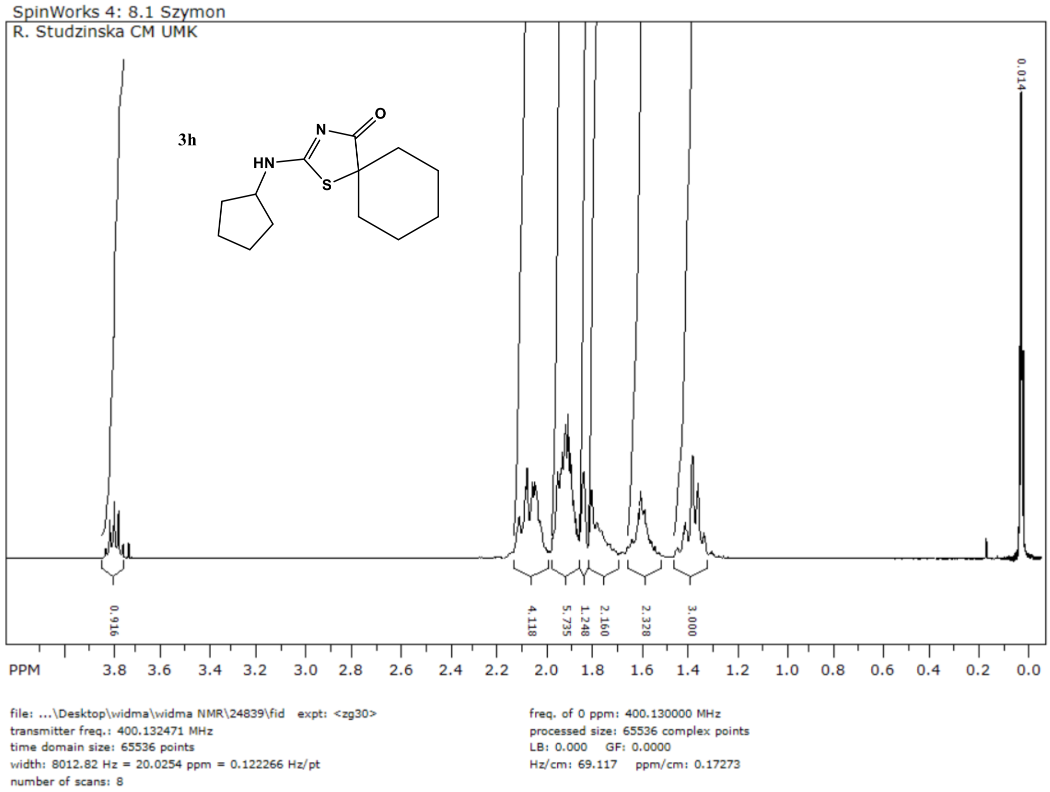

Figure S8. ^{1}H NMR spectra of compound **3h**

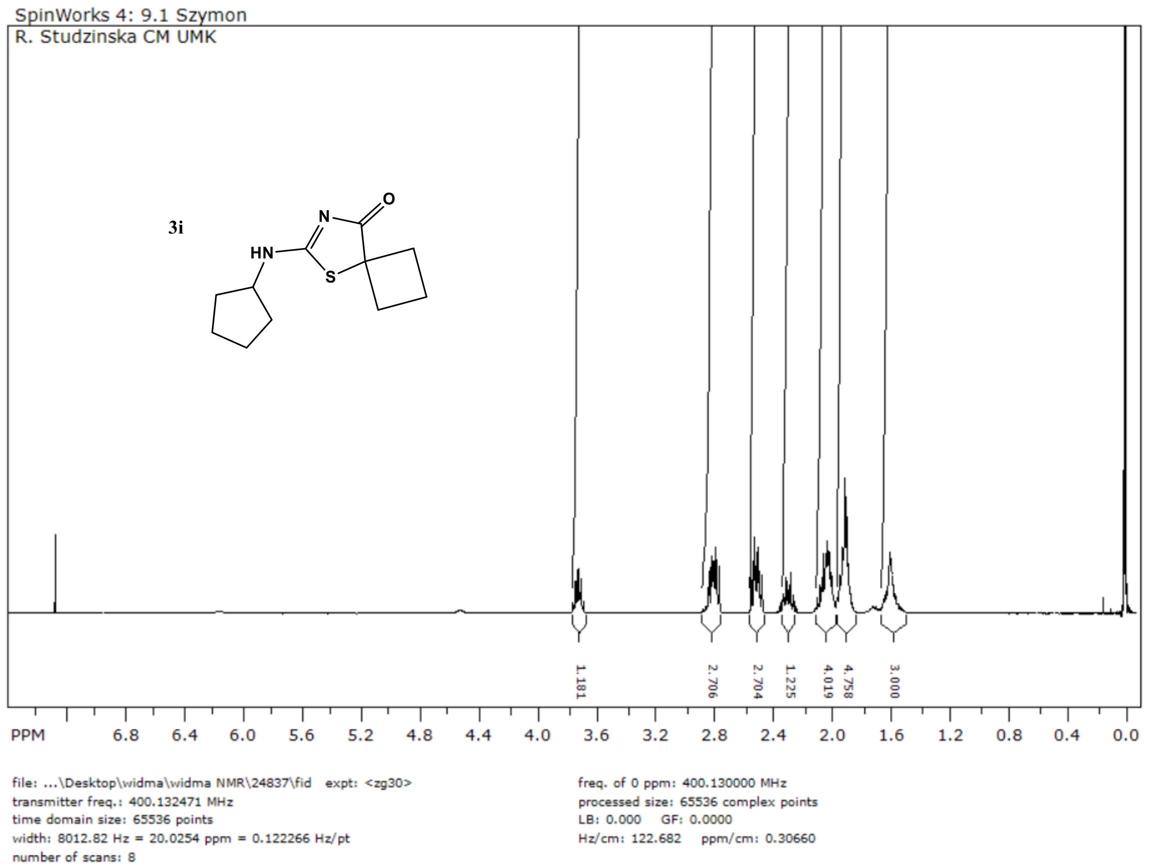

Figure S9. ^{1}H NMR spectra of compound **3i**

S2. ^{13}C NMR spectra of compounds **3a** – **3i**

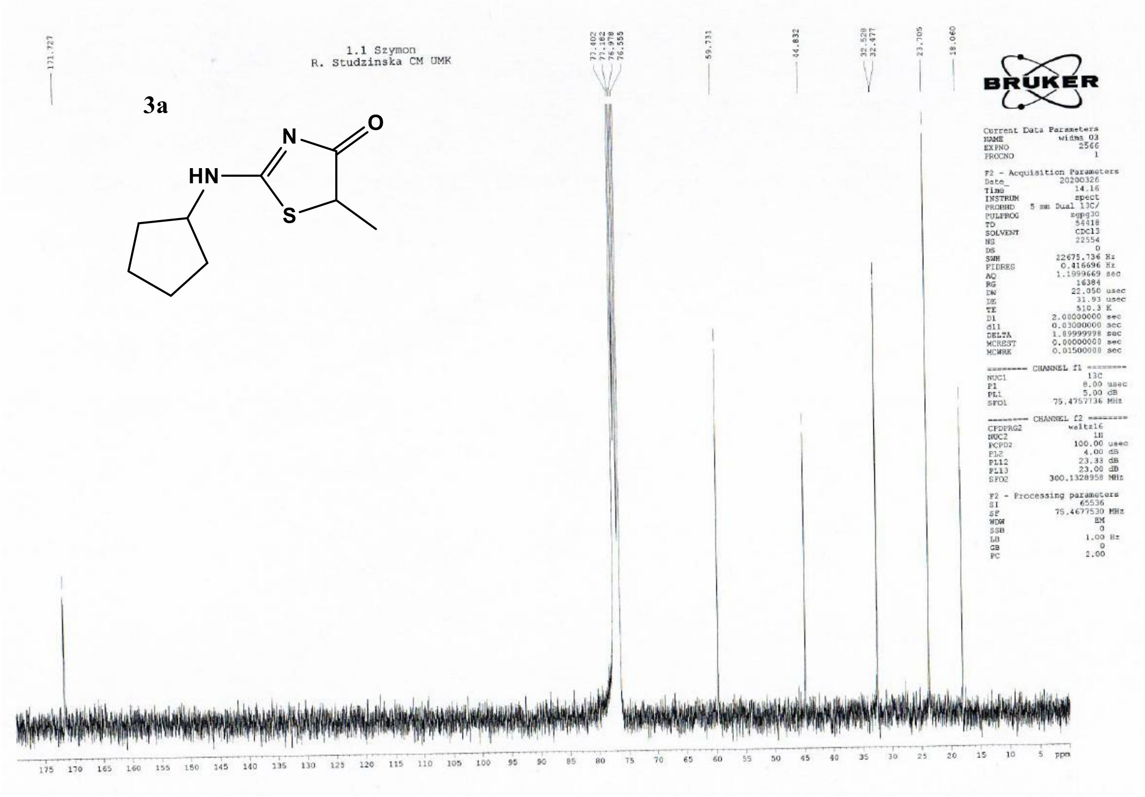

Figure S10. ^{13}C NMR spectra of compounds **3a**

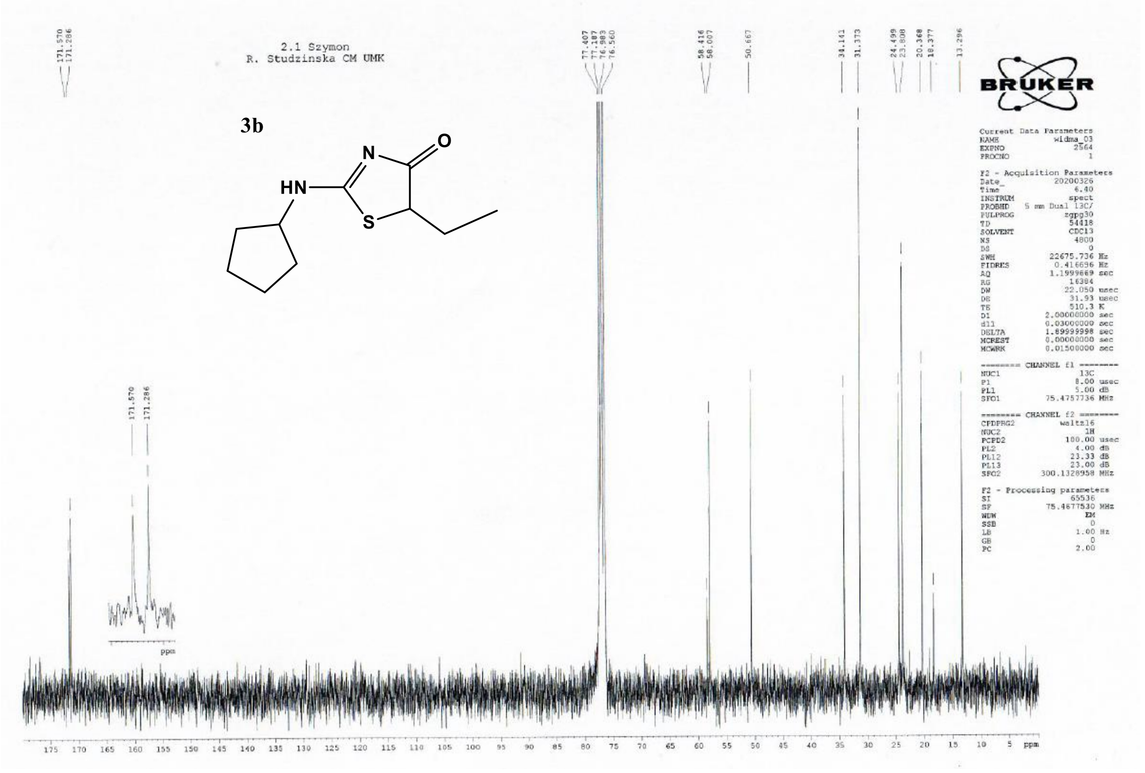

Figure S11. ^{13}C NMR spectra of compound **3b**

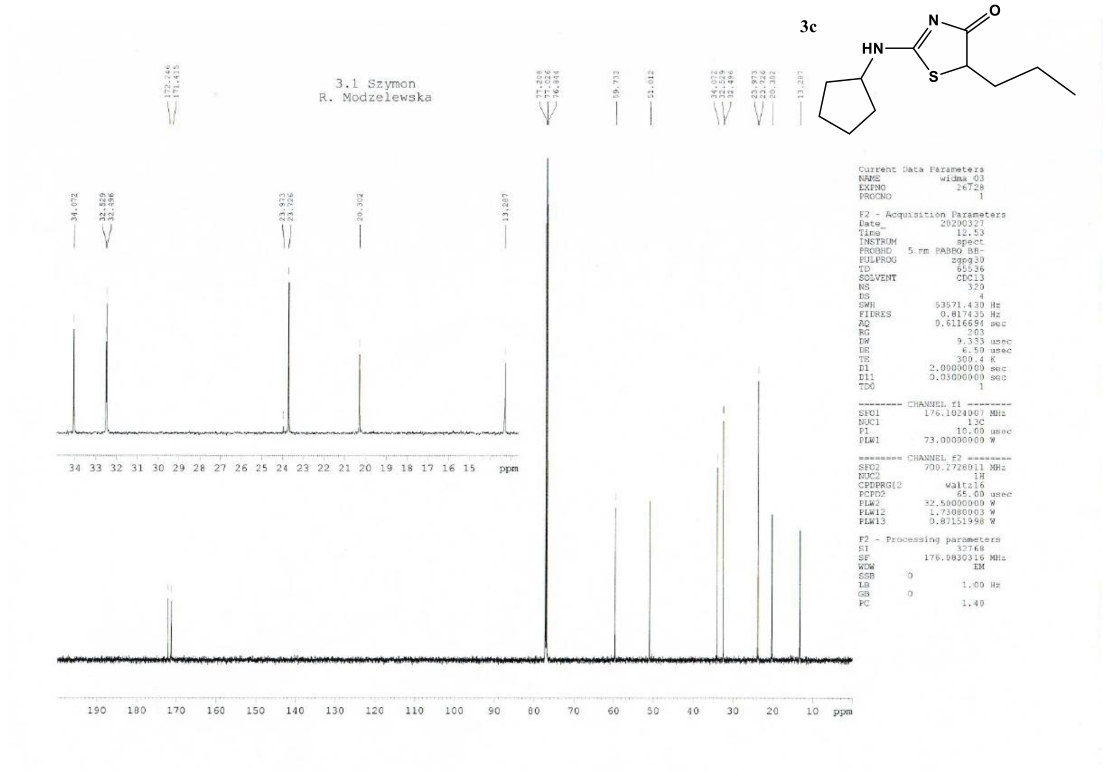

Figure S12. ^{13}C NMR spectra of compound **3c**

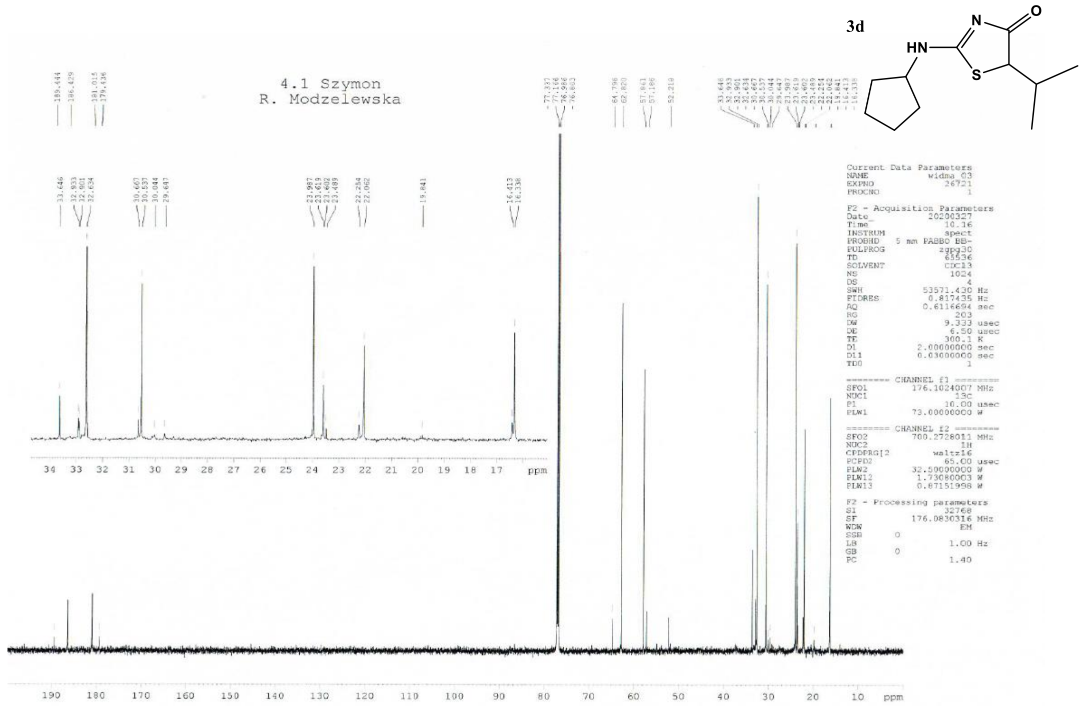

Figure S13. ^{13}C NMR spectra of compound **3d**

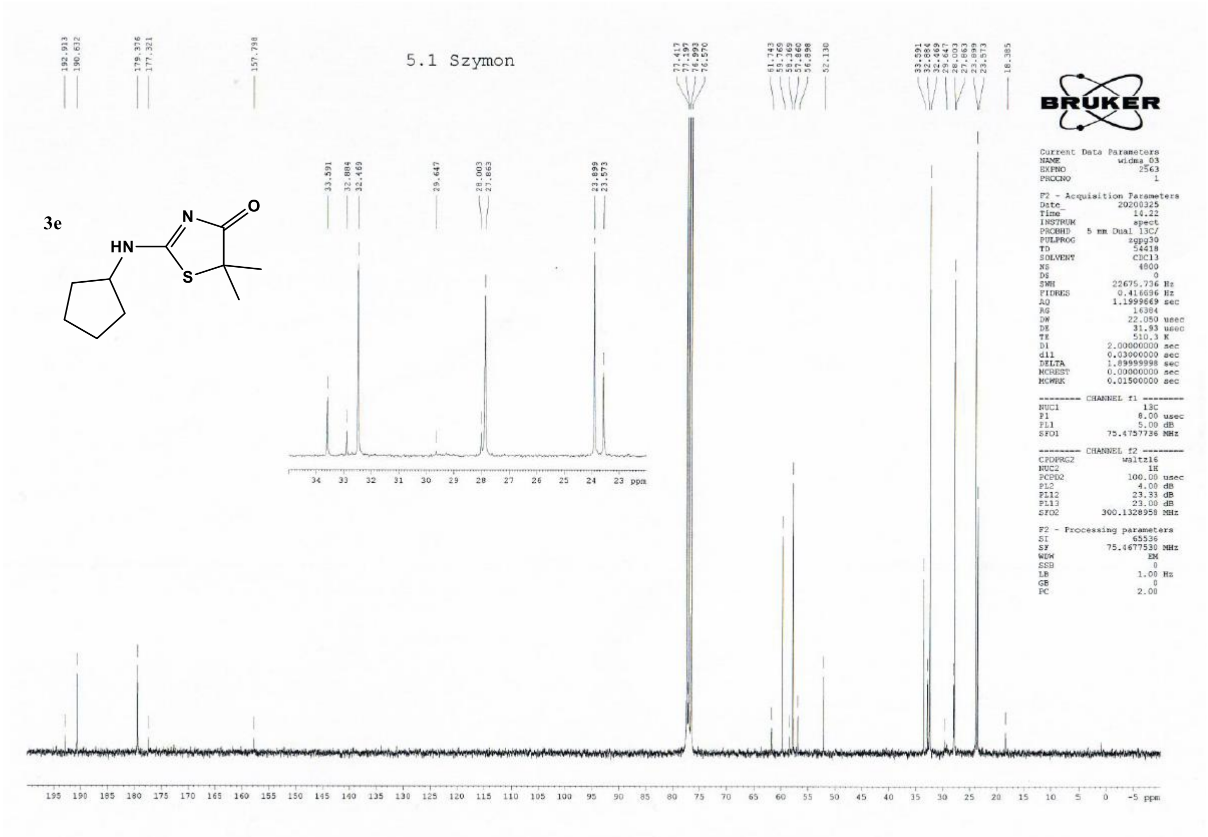

Figure S14. ^{13}C NMR spectra of compounds **3**

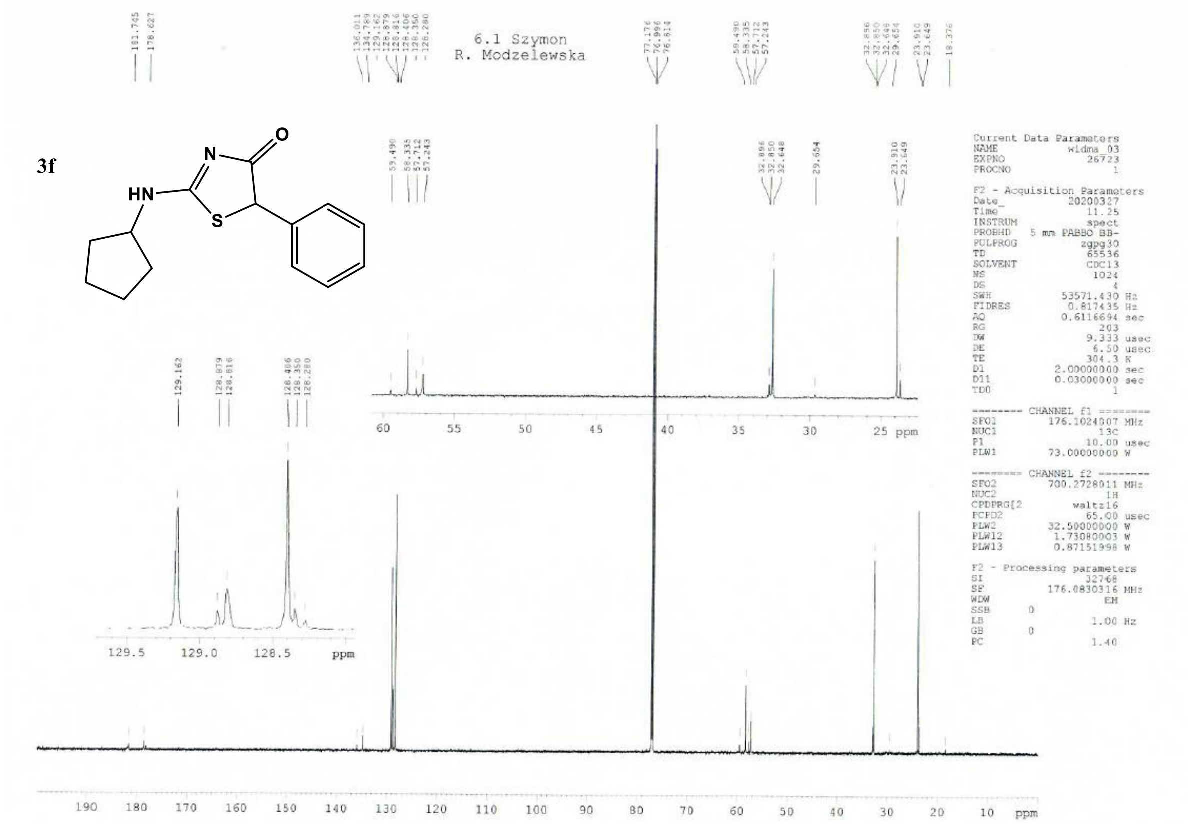

Figure S15. ^{13}C NMR spectra of compound **3f**

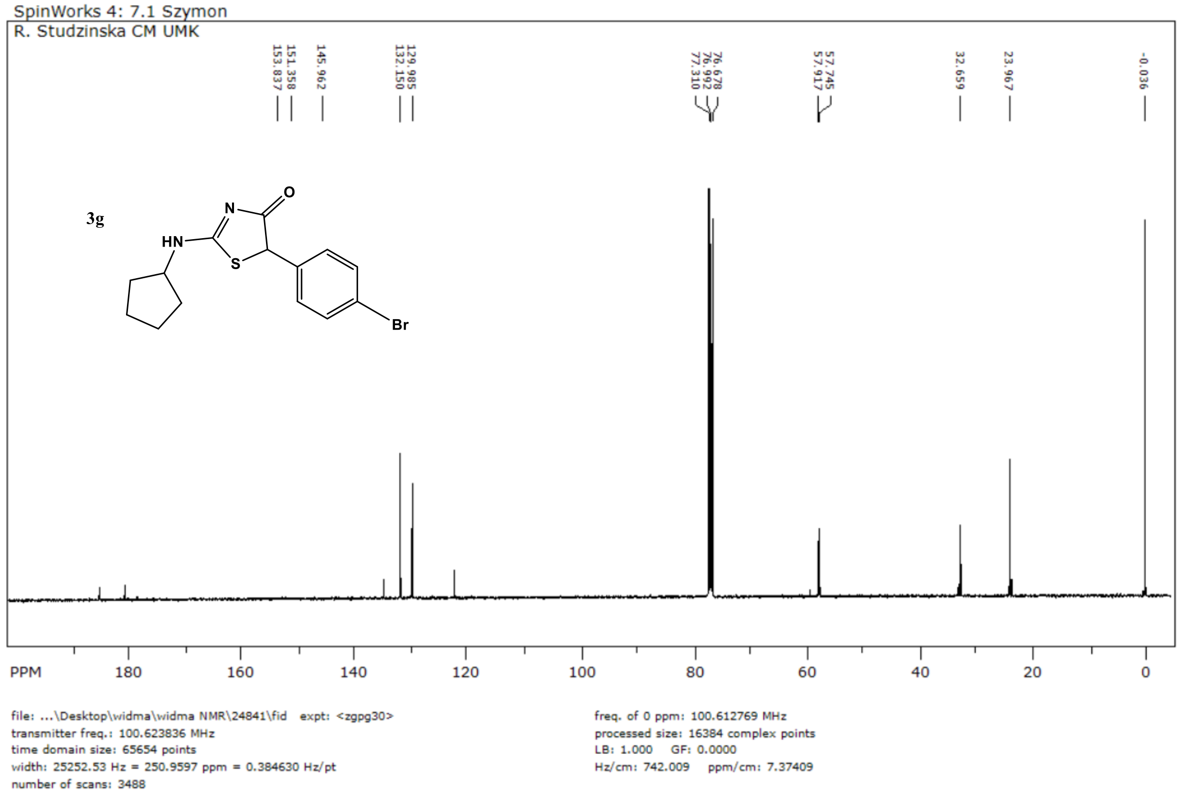

Figure S16. ^{13}C NMR spectra of compound **3g**

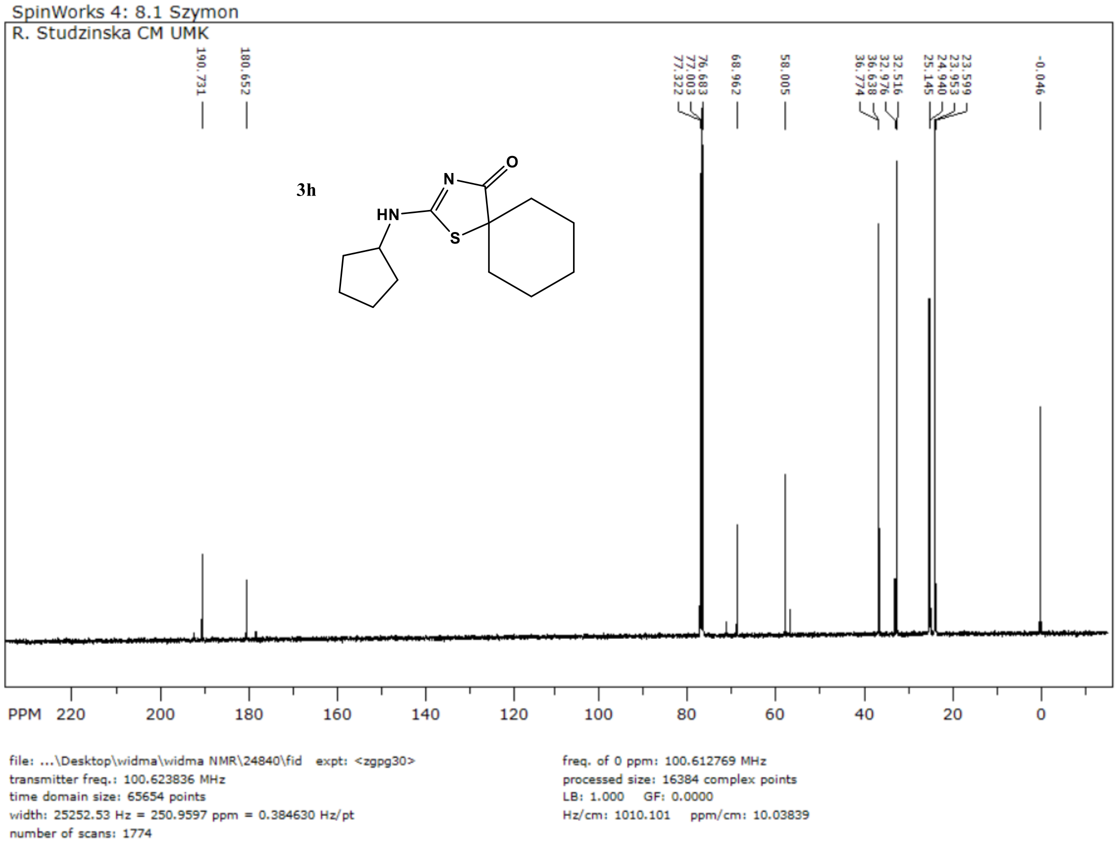

Figure S17. ^{13}C NMR spectra of compound **3h**

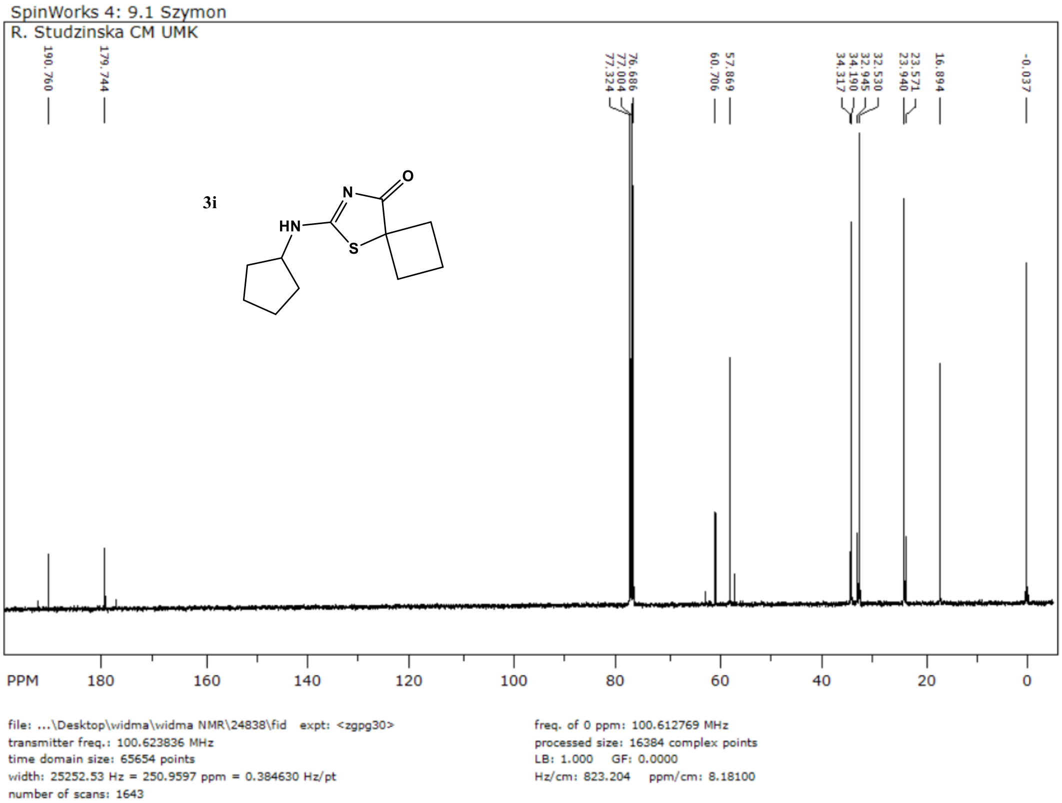

Figure S18. ^{13}C NMR spectra of compound **3i**

S3. HRMS spectra of compounds 3a – 3i

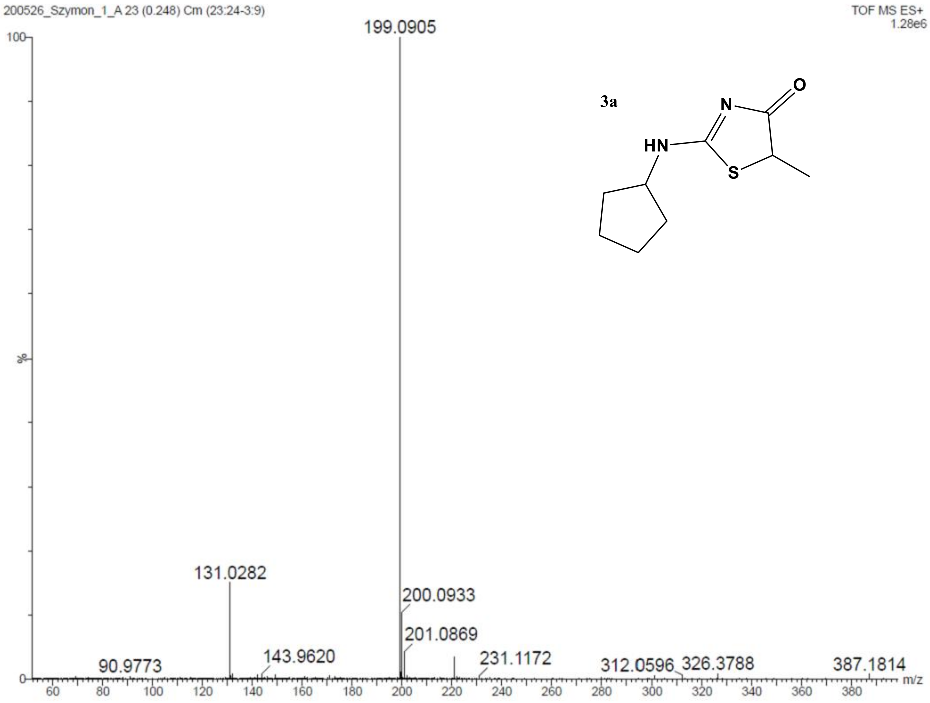

Figure S19. Mass spectrum of compound **3a**

Elemental Composition Report

Single Mass Analysis

3b

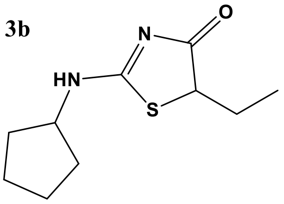

Tolerance = 10.0 PPM / DBE: min = -1.5, max = 70.0
Element prediction: Off
Number of isotope peaks used for i-FIT = 9

Monoisotopic Mass, Even Electron Ions
37 formula(e) evaluated with 1 results within limits (all results (up to 1000) for each mass)
Elements Used:
C: 0-40 H: 0-40 N: 0-2 O: 0-1 S: 0-1
200526_Szymon_2A 12 (0.143) Cm (10:12-3:8)

TOF MS ES+
6.06e+006

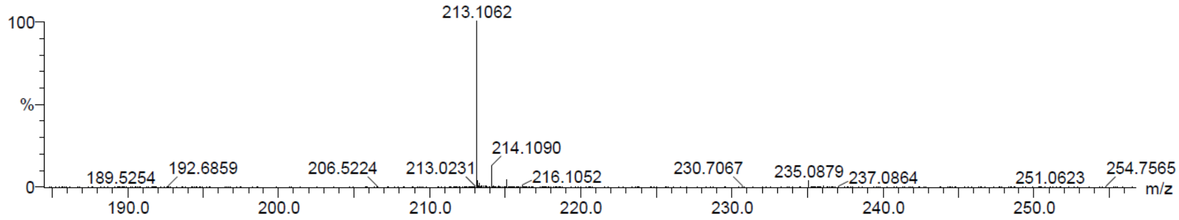

Minimum:				-1.5				
Maximum:		5.0	10.0	70.0				

Mass	Calc. Mass	mDa	PPM	DBE	i-FIT	Norm	Conf(%)	Formula
213.1062	213.1062	0.0	0.0	3.5	1191.5	n/a	n/a	C10 H17 N2 O S

Figure S20. HRMS spectra of compound **3b**

Elemental Composition Report

Single Mass Analysis

3c

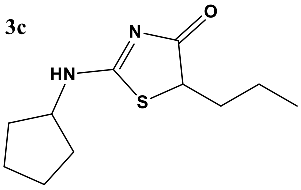

Tolerance = 10.0 PPM / DBE: min = -1.5, max = 70.0
Element prediction: Off
Number of isotope peaks used for i-FIT = 9

Monoisotopic Mass, Even Electron Ions
40 formula(e) evaluated with 1 results within limits (all results (up to 1000) for each mass)
Elements Used:
C: 0-40 H: 0-40 N: 0-2 O: 0-1 S: 0-1
200526_Szymon_3A 26 (0.285) Cm (26:32-3:8)

TOF MS ES+
6.49e+006

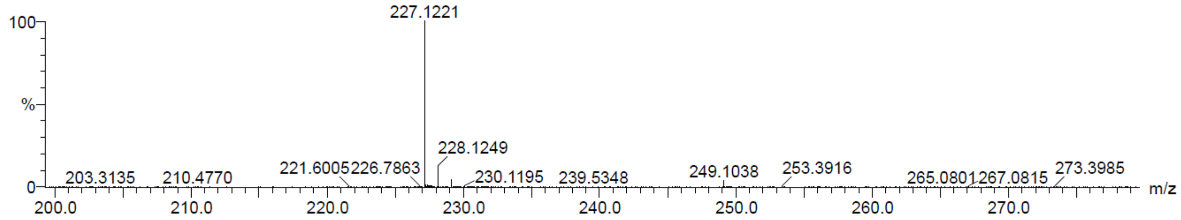

Minimum:				-1.5				
Maximum:		5.0	10.0	70.0				

Mass	Calc. Mass	mDa	PPM	DBE	i-FIT	Norm	Conf(%)	Formula
227.1221	227.1218	0.3	1.3	3.5	1214.3	n/a	n/a	C11 H19 N2 O S

Figure S21. HRMS spectra of compound **3c**

Elemental Composition Report

Single Mass Analysis

Tolerance = 10.0 PPM / DBE: min = -1.5, max = 70.0
Element prediction: Off
Number of isotope peaks used for i-FIT = 9

3d

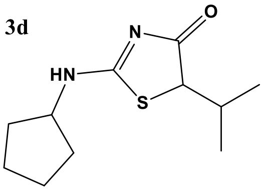

Monoisotopic Mass, Even Electron Ions
40 formula(e) evaluated with 1 results within limits (all results (up to 1000) for each mass)
Elements Used:
C: 0-40 H: 0-40 N: 0-2 O: 0-1 S: 0-1

200526_Szymon_4B 30 (0.320) Cm (30:39-2:8)

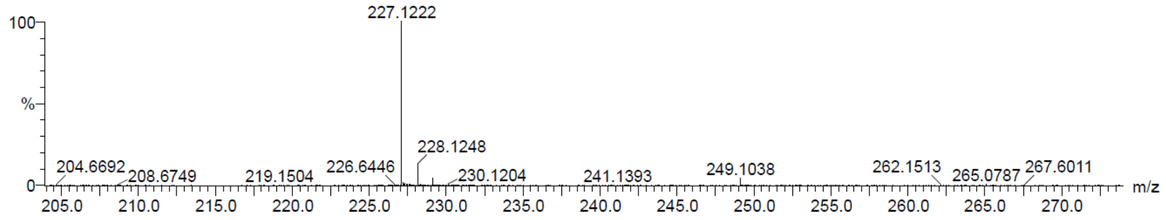

Minimum:				-1.5				
Maximum:		5.0	10.0	70.0				
Mass	Calc. Mass	mDa	PPM	DBE	i-FIT	Norm	Conf(%)	Formula
227.1222	227.1218	0.4	1.8	3.5	1458.8	n/a	n/a	C11 H19 N2 O S

Figure S22. HRMS spectra of compound **3d**

Elemental Composition Report

Page 1

Single Mass Analysis

Tolerance = 10.0 PPM / DBE: min = -1.5, max = 70.0
Element prediction: Off
Number of isotope peaks used for i-FIT = 9

3e

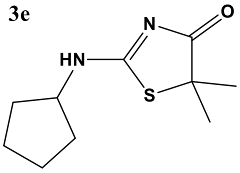

Monoisotopic Mass, Even Electron Ions
37 formula(e) evaluated with 1 results within limits (all results (up to 1000) for each mass)
Elements Used:
C: 0-40 H: 0-40 N: 0-2 O: 0-1 S: 0-1

200526_Szymon_5A 32 (0.337) Cm (31:37-2:10)

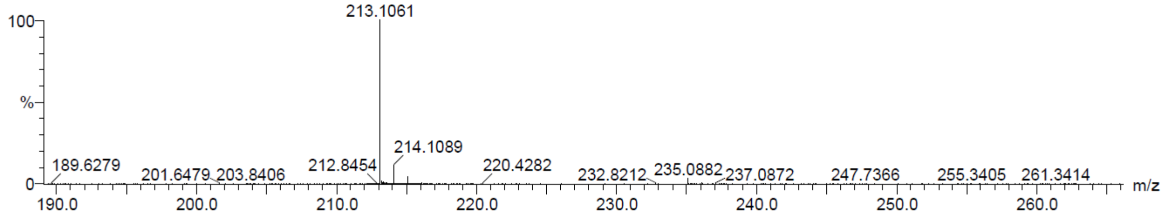

Minimum:				-1.5				
Maximum:		5.0	10.0	70.0				
Mass	Calc. Mass	mDa	PPM	DBE	i-FIT	Norm	Conf(%)	Formula
213.1061	213.1062	-0.1	-0.5	3.5	1438.4	n/a	n/a	C10 H17 N2 O S

Figure S23. HRMS spectra of compound **3e**

Elemental Composition Report

3f

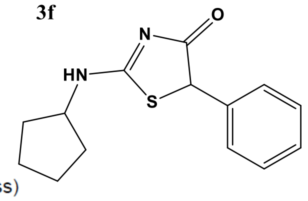

Single Mass Analysis
Tolerance = 10.0 PPM / DBE: min = -1.5, max = 70.0
Element prediction: Off
Number of isotope peaks used for i-FIT = 9

Monoisotopic Mass, Even Electron Ions
26 formula(e) evaluated with 1 results within limits (all results (up to 1000) for each mass)
Elements Used:
C: 0-20 H: 0-25 N: 0-2 O: 0-1 S: 0-1

200526_Szymon_6A 57 (0.597) Cm (57:65-3:8)
TOF MS ES+
5.95e+006

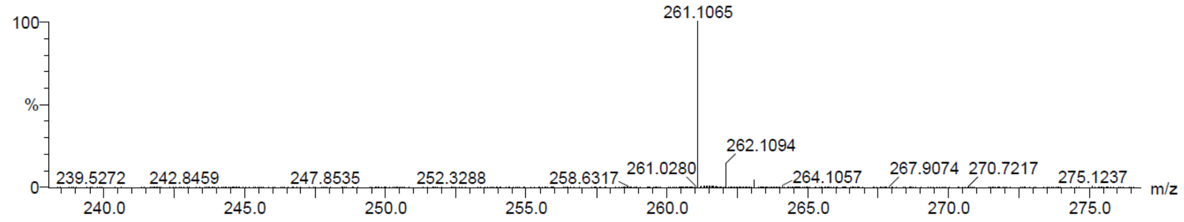

Minimum:				-1.5				
Maximum:		5.0	10.0	70.0				
Mass	Calc. Mass	mDa	PPM	DBE	i-FIT	Norm	Conf(%)	Formula
261.1065	261.1062	0.3	1.1	7.5	1603.1	n/a	n/a	C14 H17 N2 O S

Figure S24. HRMS spectra of compound **3f**

Elemental Composition Report

Page 1

3g

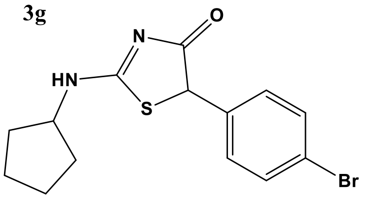

Single Mass Analysis
Tolerance = 5.0 PPM / DBE: min = -1.5, max = 80.0
Element prediction: Off
Number of isotope peaks used for i-FIT = 9

Monoisotopic Mass, Even Electron Ions
518 formula(e) evaluated with 2 results within limits (all results (up to 1000) for each mass)
Elements Used:
C: 0-60 H: 0-70 N: 0-6 O: 0-4 S: 0-1 Br: 0-1

210318_7_1_SzymonA 18 (0.205) Cm (18:30-4:8)
TOF MS ES+
1.35e+007

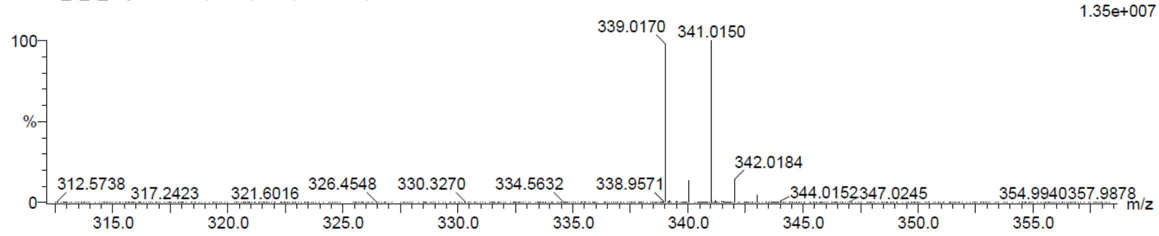

Minimum:				-1.5				
Maximum:		5.0	5.0	80.0				
Mass	Calc. Mass	mDa	PPM	DBE	i-FIT	Norm	Conf(%)	Formula
339.0170	339.0167	0.3	0.9	7.5	1318.5	0.000	100.00	C14 H16 N2 O S Br
	339.0154	1.6	4.7	19.5	1346.0	27.545	0.00	C18 H3 N4 O4

Figure S25. HRMS spectra of compound **3g**

Elemental Composition Report

3h

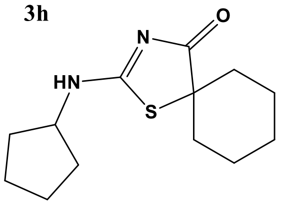

Single Mass Analysis

Tolerance = 5.0 PPM / DBE: min = -1.5, max = 80.0
Element prediction: Off
Number of isotope peaks used for i-FIT = 9

Monoisotopic Mass, Even Electron Ions
224 formula(e) evaluated with 1 results within limits (all results (up to 1000) for each mass)
Elements Used:
C: 0-60 H: 0-70 N: 0-6 O: 0-4 S: 0-1
210318_8_1_SzymonA 28 (0.303) Cm (28:40)

TOF MS ES+
2.92e+007

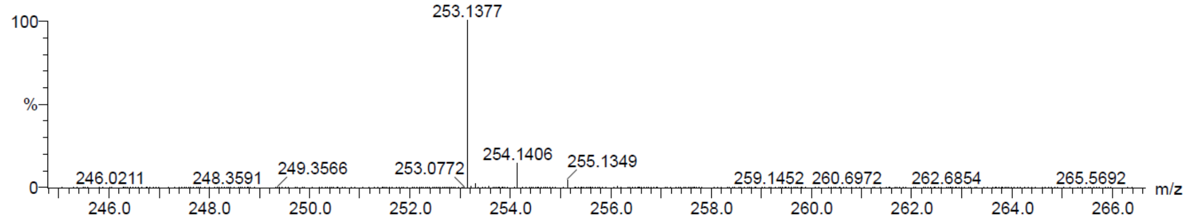

Minimum:				-1.5				
Maximum:		5.0	5.0	80.0				
Mass	Calc. Mass	mDa	PPM	DBE	i-FIT	Norm	Conf(%)	Formula
253.1377	253.1375	0.2	0.8	4.5	1308.6	n/a	n/a	C13 H21 N2 O S

Figure S26. HRMS spectra of compound **3h**

Elemental Composition Report

3i

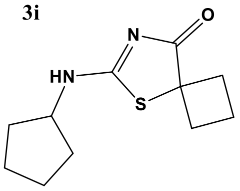

Single Mass Analysis

Tolerance = 5.0 PPM / DBE: min = -1.5, max = 80.0
Element prediction: Off
Number of isotope peaks used for i-FIT = 9

Monoisotopic Mass, Even Electron Ions
200 formula(e) evaluated with 1 results within limits (all results (up to 1000) for each mass)
Elements Used:
C: 0-60 H: 0-70 N: 0-6 O: 0-4 S: 0-1
210318_9_1_SzymonA 21 (0.231) Cm (21:36)

TOF MS ES+
3.32e+007

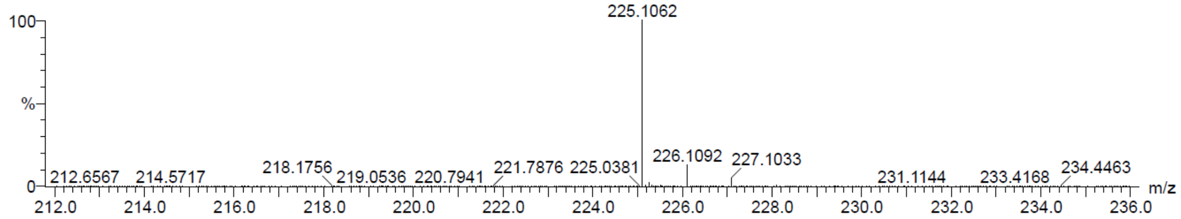

Minimum:				-1.5				
Maximum:		5.0	5.0	80.0				
Mass	Calc. Mass	mDa	PPM	DBE	i-FIT	Norm	Conf(%)	Formula
225.1062	225.1062	0.0	0.0	4.5	1472.9	n/a	n/a	C11 H17 N2 O S

Figure S27. HRMS spectra of compound **3i**

S4. Cell metabolic activity changes in normal and cancer cell lines treated with compounds **3a – 3i** (numerical data to Fig.4 of manuscript)

**Compound 3a**												
**Concentration [µM]**	**Cell line**											
	**BJ**		**Caco-2**		**PANC-1**		**U118-MG**		**SK-MEL-30**		**MDA-MB-231**	
**C**	100.0	±7.5	100.0	±9.8	100.0	±11.7	100.0	±10.1	100.0	±7.3	100.0	±7.0
**10**	95.9	±15.0	86.2	±4.8	99.7	±14.5	110.8	±4.2	103.6	±4.6	104.8	±12.2
**20**	98.4	±10.8	92.8	±6.7	103.5	±7.2	108.9	±3.5	102	±3.8	106.7	±7.5
**50**	102.4	±5.2	82.6	±9.2	105.8	±6.8	113	±6.0	101.7	±3.9	103	±4.2
**100**	111.5	±3.0	81.6	±6.6	107.1	±8.7	109.9	±4.5	106.2	±3.7	105	±10.6
**200**	109.2	±4.5	76.9	±6.7	109.7	±8.1	110.5	±4.9	102.7	±3.1	104.4	±11.9
**300**	109.2	±8.6	75.5	±8.8	105.6	±7.0	109.8	±6.5	100.8	±1.7	93.6	±9.8
**500**	110.4	±15.3	66.8	±5.5	104.1	±5.9	104.8	±9.0	97.6	±1.6	89.8	±11.9

**Compound 3b**												
**Concentration [µM]**	**Cell line**											
	**BJ**		**Caco-2**		**PANC-1**		**U118-MG**		**SK-MEL-30**		**MDA-MB-231**	
**C**	100.0	±4.0	100.0	±9.4	100.0	±16.0	100.0	±9.1	100.0	±8.7	100.0	±4.0
**10**	97.9	±2.5	87.9	±3.9	102.7	±13.5	110.0	±5.6	93.7	±15.6	97.5	±9.2
**20**	98.5	±4.2	87.3	±4.8	102.2	±10.9	107.2	±4.5	99.0	±13.8	97.8	±8.7
**50**	102.1	±3.1	84.4	±3.6	107.1	±7.3	110.7	±6.4	97.4	±10.9	94.5	±5.2
**100**	108.1	±5.1	79.8	±4.1	111.7	±6.7	112.9	±4.4	105.4	±2.4	95.3	±4.1
**200**	110.5	±5.4	77.0	±5.0	113.3	±14.1	113.3	±6.8	100.9	±3.1	86.5	±2.8
**300**	111.8	±8.7	73.2	±4.7	109.7	±10.3	112.5	±6.0	98.4	±2.8	75.3	±3.0
**500**	110.8	±11,3	66,1	±4,2	105,1	±11,3	106,6	±7,1	93,8	±6,4	57,4	±16,5

Compound 3c												
Concentration [µM]	Cell line											
	BJ		Caco-2		PANC-1		U118-MG		SK-MEL-30		MDA-MB-231	
**C**	100.0	±7.3	100.0	±9.1	100.0	±12.3	100.0	±11.3	100.0	±4.7	100.0	±5.7
**10**	98.2	±4.9	88.1	±5.2	107.8	±7.8	111.1	±4.0	100.6	±4.6	101.0	±4.1
**20**	99.7	±6.7	86.6	±3.9	105.6	±5.4	110.2	±3.3	102.0	±4.8	102.3	±3.4
**50**	102.1	±6.2	87.6	±4.4	111.5	±9.1	110.2	±3.4	100.8	±4.1	99.1	±4.0
**100**	107.6	±3.4	82.0	±5.2	114.3	±8.0	113.9	±3.1	101.0	±3.2	97.4	±2.1
**200**	112.5	±9.9	86.1	±6.0	115.9	±5.5	112.7	±3.5	95.9	±5.3	88.4	±3.0
**300**	111.2	±5.3	83.1	±4.8	115.0	±9.6	111.5	±6.1	75.0	±9.8	75.9	±3.1
**500**	115.0	±9.6	68.5	±3.1	122.1	±10.1	111.8	±14.8	45.5	±14.2	55.0	±5.2

Compound 3d												
Concentration [µM]	Cell line											
	BJ		Caco-2		PANC-1		U118-MG		SK-MEL-30		MDA-MB-231	
**C**	100.0	±9.3	100.0	±8.7	100.0	±9.3	100.0	±9.0	100.0	±6.9	100.0	±4.1
**10**	109.1	±11.5	88.8	±6.9	107.3	±13.6	118.0	±5.7	97.2	±3.6	92.8	±6.1
**20**	105.5	±5.5	82.8	±3.2	103.4	±10.0	111.1	±5.7	97.1	±1.7	91.2	±6.3
**50**	108.7	±9.0	90.8	±5.5	105.4	±9.2	114.3	±5.0	97.7	±2.6	95.5	±2.3
**100**	110.0	±3.6	83.1	±3.1	116.0	±9.7	111.9	±4.4	101.5	±2.8	94.4	±2.4
**200**	116.0	±4.7	84.8	±4.1	112.9	±5.2	113.7	±3.9	92.3	±4.2	86.6	±1.9
**300**	118.1	±5.3	85.3	±3.8	118.8	±10.1	115.1	±1.9	78.2	±2.1	81.8	±1.7
**500**	117.6	±14.6	70.1	±4.6	117.8	±18.0	120.0	±4.6	59.4	±6.6	57.6	±3.0

Compound 3e												
**Concentration [µM]**	**Cell line**											
	**BJ**		**Caco-2**		**PANC-1**		**U118-MG**		**SK-MEL-30**		**MDA-MB-231**	
**C**	100.0	±12.1	100.0	±13.1	100.0	±12.1	100.0	±10.3	100.0	±3.5	100.0	±4.0
**10**	107.2	±10.5	99.9	±4.6	110.0	±4.5	110.1	±2.7	101.5	±7.3	96.3	±1.5
**20**	109.2	±8.5	86.5	±2.8	100.9	±8.5	109.5	±4.0	100.3	±3.5	100.1	±2.4
**50**	111.5	±8.7	86.7	±3.0	109.7	±4.3	115.6	±3.8	103.0	±2.7	103.1	±2.5
**100**	114.0	±6.5	83.4	±3.7	111.7	±6.4	111.0	±5.2	106.0	±3.3	95.7	±4.5
**200**	117.3	±6.4	80.2	±2.9	120.5	±6.9	111.7	±6.5	106.3	±1.7	93.7	±2.8
**300**	118.8	±6.1	75.6	±2.6	122.2	±8.6	108.7	±8.8	104.1	±2.8	90.6	±2.5
**500**	108.2	±11.2	64.1	±2.6	127.1	±16.2	106.5	±12.1	103.9	±2.9	85.2	±3.7

Compound 3f												
**Concentration [µM]**	**Cell line**											
	**BJ**		**Caco-2**		**PANC-1**		**U118-MG**		**SK-MEL-30**		**MDA-MB-231**	
**C**	100.0	±6.9	100.0	±7.5	100.0	±14.3	100.0	±11.3	100.0	±5.5	100.0	±4.4
**10**	106.6	±11.8	91.3	±5.8	110.2	±5.5	115.8	±7.1	96.1	±4.9	96.2	±2.6
**20**	102.3	±14.0	90.4	±2.6	102.3	±9.7	114.0	±8.8	98.2	±6.3	100.5	±1.5
**50**	102.5	±4.7	93.7	±4.8	117.2	±10.4	114.3	±6.7	99.7	±3.7	95.3	±4.7
**100**	105.6	±6.0	87.4	±3.7	116.7	±13.8	115.2	±7.2	100.2	±3.2	95.9	±2.9
**200**	105.0	±5.6	89.7	±2.9	136.6	±12.5	110.1	±4.9	93.8	±5.2	87.8	±2.7
**300**	105.6	±2.6	87.1	±3.4	141.0	±13.6	105.9	±2.4	73.2	±7.0	79.1	±3.2
**500**	96.8	±10.4	75.2	±2.0	141.0	±13.9	86.8	±7.1	50.0	±2.2	78.2	±5.2

Compound 3g												
**Concentration [µM]**	**Cell line**											
	**BJ**		**Caco-2**		**PANC-1**		**U118-MG**		**SK-MEL-30**		**MDA-MB-231**	
**C**	100.0	±9.5	100.0	±10.4	100.0	±11.9	100.0	±13.2	100.0	±4.5	100.0	±4.2
**10**	99.7	±6.3	91.9	±3.9	115.6	±10.7	114.4	±12.2	95.4	±14.5	92.9	±2.4
**20**	97.8	±5.4	88.8	±3.5	121.7	±20.6	109.8	±9.5	97.5	±10.0	93.6	±2.7
**50**	90.8	±3.6	87.3	±3.8	144.9	±41.3	104.9	±6.6	98.3	±12.8	84.6	±5.2
**100**	88.1	±5.1	80.7	±4.3	139.2	±48.7	103.5	±2.2	93.1	±18.8	82.0	±1.4
**200**	84.2	±9.8	56.3	±6.8	94.1	±24.8	87.3	±5.2	71.5	±9.7	61.4	±2.2
**300**	57.3	±5.5	30.1	±5.7	57.9	±6.6	40.6	±7.1	29.7	±2.0	43.9	±0.4
**500**	55.4	±7.9	30.9	±7.2	57.2	±7.1	32.3	±2.2	25.2	±2.2	38.0	±1.5

Compound 3h												
**Concentration [µM]**	**Cell line**											
	**BJ**		**Caco-2**		**PANC-1**		**U118-MG**		**SK-MEL-30**		**MDA-MB-231**	
**C**	100.0	±7.6	100.0	±9.6	100.0	±8.0	100.0	±10.1	100.0	±6.6	100.0	±5.2
**10**	108.2	±9.4	96.3	±4.1	101.6	±8.6	111.1	±7.0	96.1	±11.4	91.6	±4.5
**20**	106.3	±8.5	94.2	±5.5	96.0	±6.8	111.3	±7.7	100.4	±9.8	92.7	±4.6
**50**	111.0	±4.0	99.9	±3.3	105.1	±6.0	116.3	±5.5	102.1	±11.6	92.1	±3.1
**100**	118.0	±10.2	96.2	±4.0	108.6	±8.3	116.2	±4.5	105.0	±11.5	93.5	±3.5
**200**	121.9	±12.5	95.6	±4.9	116.6	±8.6	121.3	±2.3	109.1	±9.5	77.1	±4.5
**300**	115.1	±20.8	82.6	±4.8	127.0	±12.6	121.1	±5.9	106.1	±6.6	64.6	±4.1
**500**	79.0	±35.6	65.5	±4.8	98.4	±21.4	99.9	±10.9	80.8	±10.8	45.6	±2.4

Compound 3i												
**Concentration [µM]**	**Cell line**											
	**BJ**		**Caco-2**		**PANC-1**		**U118-MG**		**SK-MEL-30**		**MDA-MB-231**	
**C**	100.0	±9.9	100.0	±8.9	100.0	±7.7	100.0	±9.0	100.0	±3.9	100.0	±4.2
**10**	102.4	±7.8	92.2	±4.7	95.4	±8.0	107.1	±3.6	88.1	±12.7	87.2	±2.6
**20**	103.7	±9.0	91.2	±4.0	93.3	±6.8	108.1	±3.7	94.0	±7.6	91.2	±1.4
**50**	104.7	±8.1	92.9	±5.4	97.3	±6.7	110.9	±4.5	97.3	±6.5	89.6	±1.6
**100**	110.8	±7.2	82.5	±3.0	91.4	±12.1	108.6	±4.4	99.0	±3.6	85.1	±3.3
**200**	115.7	±5.3	77.5	±3.1	91.2	±26.0	112.5	±2.8	100.7	±4.3	72.5	±9.1
**300**	121.0	±3.9	69.8	±5.3	79.7	±26.9	109.4	±3.7	102.0	±4.3	69.3	±4.7
**500**	115.4	±5.7	61.5	±2.4	66.6	±23.7	107.5	±3.7	103.1	±4.8	58.7	±4.6
